# Reconciling CD8 T cell subsets: GZMK+, cytotoxic, resident memory, exhausted, and other CD8 T cells in blood and tissue

**DOI:** 10.3389/fimmu.2026.1905956

**Published:** 2026-10-14

**Authors:** Kaylee L. Mickens, Anna Helena Jonsson

**Affiliations:** Division of Rheumatology, Department of Medicine, University of Colorado Anschutz, Aurora, CO, United States

**Keywords:** CD8 T cell, cytotoxic CD8 T cells, exhausted T cells (Tex), granzyme B, granzyme K, resident memory T (T_RM_) cells, tissue

## Abstract

CD8 T cells represent a large portion of lymphocytes in blood as well as in inflamed tissues. Recent studies have shed new light on the populations present in tissues during health and disease, prompting a re-evaluation of the roles CD8 T cells can play in human pathophysiology. Inconsistent and overlapping CD8 T cell subset nomenclature and definitions present current challenges in the field and a barrier to interpreting the literature. In this review, we discuss CD8 T cell subsets in blood and tissues, including markers, phenotypes, and migration patterns. We also address intersections among these populations, including areas of ambiguity that warrant further research. To simplify the analysis of human RNA-seq datasets from sites of disease, we propose that stratifying cells by expression of granzyme B (GZMB) versus granzyme K (GZMK) provides a useful framework that emphasizes effector function phenotypes. We lastly summarize disease associations for granzyme B- and granzyme K-expressing CD8 T cell subsets.

## Introduction

CD8 T cells are nearly as frequent as CD4 T cells in blood and most tissues, especially inflamed tissues, yet their subsets and roles in pathogenesis remain unclear in many disease settings (1–9). Some of this stems from an over-simplified view of CD8 T cell biology. Many studies assume CD8 T cells expressing any granzyme are cytotoxic and that PD-1 expression invariably indicates T cell exhaustion, but new findings from -omics and wet lab studies reveal that CD8 T cells have more diverse phenotypes and functions (1, 10–14). Conversely, the complexity of CD8 T cell subsets is also an obstacle to translational studies of human samples, where researchers may feel uncertain how to decide which genes and functional properties should define CD8 T cell clusters. This is a crucial issue given that the markers chosen as definitive for clustering can have profound impact on the interpretation of CD8 T cell roles in disease pathophysiology.

Several recent CD8 T cell reviews have focused on one particular subset, e.g., exhausted CD8 T cells or regulatory CD8 T cells (15–20). These in-depth reviews are helpful in presenting detailed information about the markers and functions of that particular CD8 T cell subset, but they do not provide guidance for researchers faced with the task of classifying CD8 T cells in RNA-sequencing (RNA-seq) data or other high-parameter datasets. In this review, we address this gap by discussing common classification systems and CD8 T cell subsets, including their main effector functions and their relative frequencies of these subsets in blood and tissues. We also address areas of overlap and ambiguity between these different CD8 T cell subsets.

Secondly, we present a simplified framework that focuses on CD8 T cell effector functions by stratifying CD8 T cells by expression of GZMB and GZMK. Despite their similar names, these two proteases have quite different effects on target cells and are markers of distinct effector function programs that can be applied to quickly characterize CD8 T cells (1, 21). As new datasets from healthy and diseased human tissues are being collected at an ever-increasing pace, correctly identifying CD8 T cell subsets in -omics datasets is crucial to accurately interpreting the roles of these cells in disease pathophysiology. The goal of this review is to provide a nuanced understanding of CD8 T cell subsets in blood and tissues that can be applied to the growing number of single-cell RNA-seq and other -omic datasets of human patient samples in infections, cancers and autoimmune diseases.

## Overview of CD8 T cell populations in healthy blood

CD8 T cells in blood are a mixture of naïve and memory cells. The easiest way to subset circulating CD8 T cells relies on surface proteins CD45RA, CD45RO, and CCR7 (in humans) to classify cells as naïve (CD45RA+ CD45RO- CCR7+), central memory (T_CM_, CD45RA- CD45RO+ CCR7+), effector memory (T_EM_, CD45RA- CD45RO+ CCR7-), and terminally differentiated effector memory cells (T_EMRA_, CD45RA+ CD45RO- CCR7-) (22, 23). This classification system works well for flow cytometry and other protein-based techniques (including CITE-seq), but it is difficult to use in purely mRNA-based datasets since CD45RA and CD45RO are alternative splicing products of the same gene (*PTPRC*).

### Naïve CD8 T cells

The majority of CD8 T cells in blood of children and young adults are naïve, meaning that they have not encountered their cognate peptide antigen (24–26). These cells express CD45RA; CCR7, a chemokine receptor that directs cells to enter lymph nodes; and CD62L, an integrin that allows these cells to adhere to and translocate across high endothelial venules in secondary lymphoid organs (23). Naïve CD8 T cells circulate through lymph nodes searching for their cognate antigen presented by antigen-presenting cells (APCs).

### Central memory, effector memory, and T_EMRA_ CD8 T cells

Central memory T (T_CM_) cells express CD45RO, CCR7, and CD62L. The term “central” comes from their ability to traffic through lymph nodes via expression of CCR7 and CD62L. In contrast, effector memory (T_EM_) T cells do not express CCR7 or CD62L. Instead, CD8 T_CM_ traffic through peripheral sites of inflammation via their expression of other chemokine receptors, such as CCR2, CCR5, and CXCR3 (23, 27). This population is generally considered to consist of short-lived cells that clear up remaining infected cells after an infection and then die away (23). T_EMRA_ are effector memory CD8 T cells (i.e. they lack CCR7 and do not traffic through lymph nodes, similar to T_EM_) that have re-expressed CD45RA (18). These cells are considered terminally differentiated, and they tend to express cytotoxic proteins such as GZMB and perforin (28, 29).

### T stem cell memory CD8 T cells

T stem cell memory CD8 T cells (T_SCM_) are a fourth memory cell subset that can be misassigned to the naïve cell compartment when cells are classified solely by CCR7 and CD45RA/CD45RO. CD8 T_SCM_ cells express CD45RA and CCR7, like naïve CD8 T cells, but differ from naïve cells in that they also express CD95 (FAS) (23, 30). Their name comes from their ability to self-renew, proliferate, and differentiate into many other CD8 memory T cell subsets (31, 32). T_SCM_ have the capability of extremely long term self-renewal and memory—longer than 25 years (33). T_SCM_ are mainly present in blood and secondary lymphoid organs and are rare in tumor microenvironments and other tissue settings, but they can differentiate into CD8 T cell subsets such as T_CM_, T_EM_, tissue resident memory (T_RM_), and CD8 cytotoxic T lymphocytes (CD8 CTL) that are commonly found in tissues (32, 34, 35).

CD8 T_SMC_ are nicely characterized in studies of yellow fever virus (YFV) vaccination. Because YFV is not endemic in the continental United States, YFV vaccination provides a unique opportunity to study CD8 T cell phenotypes and effector functions over time after a single transient antigen exposure. In these studies, YFV-specific CD8 T cells, identified using MCH class I tetramers loaded with immunodominant YFV peptides, exhibit a cytotoxic effector phenotype in the initial weeks after vaccination (36). However, by 2 years after vaccination, many of the YFV-specific CD8 T cells revert to a “naïve” surface marker phenotype, expressing the prototypical naïve markers CD45RA and CCR7 (36). Despite this surface marker expression pattern, these YFV-specific CD8 T cells respond quickly to restimulation, even eight years after vaccination, a core functional characteristic of memory CD8 T cells (37).

### Factors influencing the frequency of CD8 T cell subsets in blood

The frequency of these different CD8 T cell subsets among circulating CD8 T cells vary from person to person depending on their respective histories of exposures and infections. The frequency of memory cells, particularly T_EMRA_, goes up with age (38), and individuals with chronic active infections such as HIV-1 also tend to express a higher frequency of T_EMRA_ cells (39). T_EMRA_ are often oligoclonal, meaning that many CD8 T_EMRA_ cells carry identical TCR sequences and thus belong to the same TCR clonotype (40). These expanded T_EMRA_ clonotypes are often CMV- or EBV-reactive, and this oligoclonality increases with age (41, 42). The reasons for this shift are not known, but this phenomenon is the reason why it is important to match or control for the age of participants in any studies comparing CD8 T cells in different cohorts.

## Overview of CD8 T cells in tissue:

The vast majority of CD8 T cells in the body are not found in the circulation but are instead located in tissues (43, 44). CD8 T cell subsets in tissues, especially inflamed tissues, differ significantly from the populations commonly found in blood. While the CD45RA/CD45RO/CCR7 classification system is sometimes applied to tissues, it is not as informative for cells collected from sites of disease because it does not capture the biologically relevant phenotypes of CD8 T cells in inflamed or otherwise diseased tissues. Here, we summarize these tissue-predominant CD8 T cell subsets, including markers and effector functions. Importantly, these CD8 T cell subsets are not mutually exclusive, meaning that a particular CD8 T cell may be classified in different ways depending on the type of technology used (e.g., flow cytometry versus RNA-seq) or the overall focus of the study (e.g., cancer studies may be more likely than an autoimmunity study to look for and identify certain CD8 T cells as exhausted). Furthermore, some of the cell categories have overlapping gene signatures, making full separation difficult, such as with T_RM_ and exhausted T cells (T_EX_) (30). The details of how these subsets relate to each other, such as their ontogeny and whether CD8 T cells can convert from one subset to another, are ongoing areas of investigation.

### Tissue resident memory CD8 T cells

Some CD8 T cells transiently traffic through tissues in response to cytokine and chemokine signals that have recruited them to active sites, such as infection or malignancy. In contrast, CD8 tissue-resident memory T (T_RM_) cells reside in tissues permanently, without recirculating (30, 45). CD8 T_RM_ comprise a large portion of CD8 T cells in multiple tissues at rest, including the lungs, intestine, skin, liver, female reproductive tract, kidney, pancreas, heart, lymph nodes, brain, salivary glands, thymus, and spleen (46–51).

Evidence for CD8 T_RM_ cells comes from experiments using parabiotic mice (52, 53), assays using IV isotope dilution (54, 55), and studies of skin grafts or organ transplants (56–58). These studies show that many T cells in the tissue did not circulate in the blood, at least during homeostatic conditions. For example, in human organ transplants, including liver, lungs, kidney, or small intestine from HLA-mismatched donors, T cells from the donor are retained in transplanted tissues for up to a decade post-transplantation (48–51, 59).

The primary function of CD8 T_RM_ is to recognize and rapidly respond to specific peptide antigens, initiating an immune response in the tissue in which they are located. CD8 T_RM_ can produce IFNγ, which facilitates recruitment of immune cells from the periphery to the site of infection (60). IFNγ can induce chemokine (CXCL9 and CXCL10) and adhesion molecule (VCAM-1) expression in tissues allowing for additional immune cell recruitment for pathogen control (61–63). CD8 T_RM_ can also directly recruit circulating immune cells to the tissue by producing chemokines themselves such as CCL3, CCL4, and XCL1 (64, 65). All these mechanisms serve to launch immune responses to repeated exposures to the same antigen.

However, CD8 T_RM_ cells can also be pathologic. For example, CD8 T_RM_ cause fixed drug eruptions, a localized rash that recurs in the same place every time an individual is exposed to a particular medication, and they may also be responsible for the fact that people with autoimmune arthritis tend to flare in the same joints (66, 67).

Unfortunately, in most cross-sectional human studies, distinguishing non-trafficking CD8 T_RM_ from newly arrived, transiently present CD8 T cells, is challenging, especially in inflamed tissues. No single definitive marker (extracellular or intracellular) described to date is present on every CD8 T_RM_ and absent on every non-tissue resident cell. Instead, most studies rely on a combination of variably specific markers (or, more recently, gene sets (10, 11)) to classify cells as CD8 T_RM_ (**Figure 1**).

**Figure 1:**
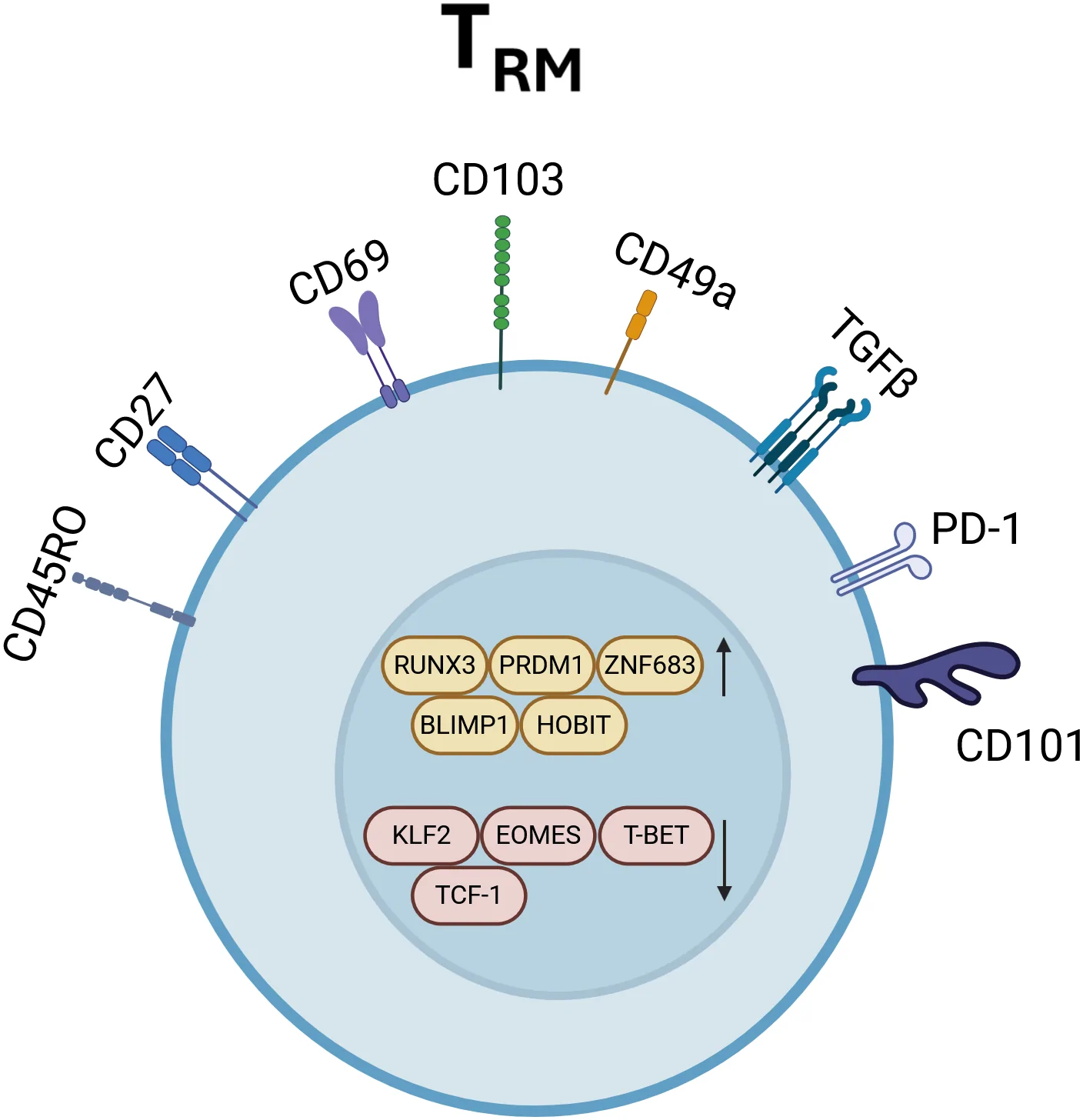
Tissue resident memory (T_RM_) CD8 T cell markers.

CD69 is one of the most commonly used of these markers (**Figure 1**). It is a sensitive but non-specific marker of CD8 T_RM_. Surface expression of CD69 in human tissue is often nearly ubiquitous in the intestines and present on a large majority of CD8 T_RM_ in the lungs, liver, kidney, and skin (48–51, 68). CD69 plays a direct role in retaining CD8 T cells in tissues by preventing the cells from responding to sphingosine-1-phosphate (S1P) (69–71). S1P is abundant in the bloodstream and acts as a chemoattractant for lymphocyte egress into circulation when bound to S1PR1. When CD8 T_RM_ express CD69, sphingosine-1-phosphate receptor (S1PR1) is internalized from the cell surface and degraded, resulting in tissue retention of the CD8 T_RM_ (69–71). On the other hand, CD69 is also a marker of recent TCR or cytokine stimulation (72). Thus, CD69 does not distinguish between CD8 T_RM_ and transiently present, activated CD8 T cells in inflamed tissues. This is a considerable problem when evaluating cells at sites of inflammation, where cells are exposed to abundant cytokines. For this reason, CD69 should not be used as the sole marker to identify CD8 T_RM_, especially in inflamed tissues.

Another marker commonly used to define CD8 T_RM_ is CD103, also known as αE integrin or ITGAE (**Figure 1**). CD103 binds E-cadherin, an epithelial cell protein, and this binding promotes tissue retention of CD8 T cells (73–75). E-cadherin expression is especially high at epithelial barrier tissue sites, so it is perhaps not surprising that CD103 expression is high among CD8 T_RM_ in the small intestine and lungs but less prominent among CD8 T_RM_ in the brain (76, 77).

CD49a (a1B1/VLA-1) is another integrin commonly expressed by CD8 T_RM_ (**Figure 1**). CD49a binds collagen IV present in the basement membrane of epithelial and endothelial tissues (78–80). CD49a expression on CD8 T_RM_ is seen in human tissues at varying degrees, with highest expression in lung, small intestine, skin, and liver (45, 49, 80–82). Among CD8 T_RM_ in the lung, CD49a engagement inhibits apoptosis and improves CD8 T cell survival (83). In skin, CD49a expression demarcates a specific subgroup of CD8 T_RM_ that localize to the basal layer of the epidermis, produce IFNγ, and express high levels of cytotoxic effectors upon stimulation (80). In contrast, CD8 T_RM_ lacking CD49a preferentially localize to the dermis and express IL-17 (80).

TGFβ signaling may be important in CD8 T_RM_ development since it induces expression of multiple tissue retention markers, including CD69, CD103, and CD49a (84–87). CD103+ T_RM_, in particular, require TGFβ signaling for normal development (88).

Some CD8 T_RM_ cells express inhibitory receptors such as PD-1 and CD101 (**Figure 1**) (89, 90). These inhibitory receptors appear to temper CD8 T_RM_ responses in tissues sensitive to inflammation (such as the pancreas) and thus contribute to protection from tissue damage (89, 91). As such, CD8 T_RM_ cells are not exhausted in the pathologic sense seen in cancer but have reduced effector functions in order to maintain homeostasis.

Several markers of CD8 T_RM_ are specific to certain tissues, indicating that CD8 T_RM_ adapt to the tissue in which they reside (45, 92). Furthermore, a given tissue may contain several CD8 T_RM_ subsets. For example, in the gut, CD8 T_RM_ are classified in two distinct groups, progenitor and differentiated, which have distinct spatial residences and effector functions (13). Specifically, progenitor CD8 T_RM_ are present in the lower villus and express *Tcf7* and *Slamf6*, while differentiated CD8 T_RM_ inhabit the upper villus and express *Gzma*, *Gzmb*, and *Itgae* (13).

A small proportion of CD8 T_RM_ can exit tissues and enter the circulation while retaining some tissue residency markers (namely CD103). These cells are called ex-T_RM_ and were recently reviewed elsewhere (93). These ex-T_RM_ can re-differentiate into circulating memory and effector cells. Other ex-T_RM_ circulate and then re-enter a different tissue location, where they revert to a T_RM_ phenotype (94). Trafficking of T_RM_ from the lung and back into the draining lymph node, where the initial immune response began, has been termed retrograde migration (95). There are many remaining questions about ex-T_RM_, including whether all T_RM_ are capable of egress, what triggers tissue egress, and when ex-T_RM_ are protective versus detrimental.

### Cytotoxic CD8 T cells

The classic effector function of CD8 T cells is to kill target cells that have been infected with intracellular pathogens (e.g., viruses) or transformed into malignant cells (**Table 1**). This effector function is primarily carried out by cytotoxic T lymphocytes (CTL), which are CD8 T cells that express high levels of GZMB, perforin, and granulysin (**Figure 2**). CD8 CTL are often called T effector cells (T_EFF_), but this designation is misleading since other T cell subsets also have effector functions. CD8 CTL killing of target cells is triggered by TCR engagement with MHC class I presenting the CTL’s cognate peptide (96). For CD8 CTL, only 2–3 TCRs on a given CD8 CTL need to engage the cognate peptide-MHC class I complex to trigger lytic granule release (97, 98). This stimulus induces formation of an immunological synapse, the molecular interface between the CD8 CTL and the target cell (99, 100). The connections of the synapse are composed of stimulatory and adhesion molecules (LFA-1, TCR, CD28, CD2) on the CD8 CTL engaging with their ligands and binding partners (ICAM-1, pMHC, CD80, CD58) on target cells (101, 102). After target cell connection and creation of the immunological synapse, the CD8 CTL then releases cytotoxic granules containing GZMB, perforin, granulysin and other cytotoxic molecules. Perforin multimerizes to form pores in the target cell membrane that allows the other granule components to enter the target cell cytoplasm to activate apoptotic pathways, including caspase cascades (103, 104). In target cells, GZMB cleaves multiple cellular targets that result in apoptosis/cell death. These targets include caspases (specifically caspases 3, 6, 7, 9), Bid, and ICAD, among others (105–107). CD8 CTL can also induce target cell death via Fas/FASL, TNFR1/TNF, TRAILR1/2 signaling.

**Table 1:** GZMB+ CD8 T cells .

Condition	Tissue	Detected as	Method	Notes	Refs
Infectious disease					
Cytomegalovirus *	blood	transcript, protein	SC, FC	CMV-specific CD8 T cells are GZMB+ in individuals with a high percentage of CMV-specific CD8 T cells	(303)
Human immunodeficiency virus *	large intestine, blood	protein	FC	GZMB+ CD8 T cells are more frequent in both blood and colon tissue of people with HIV-1, 80% of CD8 T cells reactive to Gag and Nef viral proteins express GZMB, CD8 T cells from elite controllers express GZMB and perforin more frequently than progressors, during HIV-1 infection, CD8 T cells specific for HCMV more frequently express GZMB	(39, 216–220)
Influenza *	lung, blood	transcript, protein	FC, IHC, GA	GZMB CD8 CTL are protective of severe influenza A when co-expressing GZMB and perforin	(206, 208–211, 304)
Respiratory syncytial virus *	lung	protein	IHC, FC	RSV-specific CD8 T cells are enriched in lung tissue over blood and express more GZMB after viral infection	(212–214, 305)
Herpes simplex virus-1 *	nerve (trigeminal ganglia), blood	transcript, protein	IHC, IF, GA, RT	GZMB cleaves immediate early HSV-1 protein ICP4 and limits viral reactivation	(306–308)
Herpes simplex virus-2 *	female reproductive tract, blood	protein	MSD	Transition from viral expansion to contraction is associated with GZMB expression level, during viral reactivation GZMB levels in secretions rise 1-2log	(309, 310)
SARS-CoV-2 *	blood, nasal mucosa	protein	FC	>50% CD8 T cells express GZMB and perforin from mild/asymptomatic and only 3-12% co-express in moderate/severe cases	(311, 312)
Cancer					
Non-small-cell lung cancer (NSCLC) *	lung	transcript, protein	IHC, B, FC	T_RM_ (CD103+) are enriched for GZMB expression and correlate with better survival, lower CD8 GZMB expression associate with worse progression free survival, High GZMB expression associates with better overall survival and disease-free survival	(225–228)
Breast cancer *	breast	protein	IHC	In triple-negative breast cancer, high frequency of CD8 T cells expressing GZMB in tumor tissue is associated with higher overall survival and disease-free survival	(223)
Cervical cancer *	cervical tumors	transcript	SC	CXCL13+ GZMB+ CD8 T_RM_ associate with better overall survival and progression-free survival following radiotherapy	(313)
Cutaneous melanoma*#	skin	protein	IHC, IF	Absence of GZMB+ CD8 T cells associates with metastasis to sentinel lymph nodes, however the number of GZMB+ CD8 T cells increase as tumor stage worsens and associate with poor recurrence free survival	(314, 315)
Colorectal cancer *	colorectal tumor	protein	IHC	CD8 T cell and GZMB protein presence in tumor tissue associate with all cause and CRC specific survival, positive prognosis for overall survival is associated with CD57, GZMB, and CD8, cancer cell expression of SerpinB9 (GZMB inhibitor) associates with lymph node metastasis	(221, 222)
Gastric cancer *	gastric tumor	protein, transcript	SC, IF	Abundant GZMB+ CD8 T cell tumor infiltration associates with higher overall survival in four cohorts	(224)
Hepatocellular carcinoma *	liver, blood	protein	FC, IHC	Frequency of GZMB expression in CD11c+ CD8 T cell in blood (but not tumor tissue) correlates with lower tumor volume, and frequency is higher in blood from early-stage patients than late-stage	(316)
Autoimmune					
Type 1 Diabetes Mellitus #	pancreas, blood	protein	IF, FC	Pancreas tissue contains auto-reactive CD8 T cells that increase in number as diabetes develops, CD8 T cells support diabetes development in multiple ways (direct cell killing through cytotoxic degranulation of GZMB, and cytokine secretion of TNF and IFNγ)	(235, 236, 317)
Sjogren’s Disease #	salivary gland	transcript	SC	GZMB+ GNLY+ CD8 T cells are expanded in multiple autoantibody+ subtypes and most clonally expanded subset	(318)
Systemic Lupus Erythematosus #	blood, kidney	protein, transcript	SC, FC	Increased blood CD8 T cells expressing GZMB and perforin correlates with disease activity, GZMB and GZMK CD8 T cells are enriched in kidney biopsies compared to healthy controls	(172, 319)
Multiple Sclerosis #	brain	protein	IHC	GZMB+ CD8 T cells are present in MS brain lesions and are higher in frequency in acute/relapsing-remitting MS	(320)
Susac Syndrome #	brain, blood	protein	FC, IHC, IF	GZMB+ CD8 T cells accumulate in damaged microvessles of brain in SuS patients and are more frequent in PBMC, brain endothelial cell apoptosis in SuS also associates with polarization of cytotoxic granules in CD8 T cells	(321)
Ankylosing Spondylitis	blood	transcript, protein	RT, FC	CD8+ T cells express GZMB more frequently in AS over healthy controls and GZMB negatively correlates with multiple clinical markers of AS	(322, 323)
Vitiligo #	skin	transcript, protein	B, FC	CD8 T cells in vitiligo lesions more frequently express GZMB and perforin than healthy donor skin	(80)
Crohn’s Disease #	ileum, blood	protein	FC	GZMB+ CD8 T cells are higher in blood and ileum from recurrent patients compared to those in remission	(324)
Systemic sclerosis interstitial lung disease #	lung, blood	transcript	SC	CD57+ T_EM_ associated with lung injury, express elevated levels of GZMB	(325)
Immune Checkpoint Inhibitor Colitis #	colon	transcript	SC, IMC	CD8 T cells express higher levels of GZMB in ICI colitis compared to healthy controls and other kinds of colitis	(299, 300)
Other					
Transplant rejection #	lung, skin, heart, kidney	transcript, protein	FC, B, SC	CD8 upregulate GZMB during rejection of liver grafts, CD8 T cells with high GZMB expression associate with kidney transplant pathology ranging from graft dysfunction to rejection	(237, 239, 241, 326–328)

*Favorable or #unfavorable prognostic associations with GZMB CD8 T cells; SC, scRNAseq; B, bulk RNAseq; FC, flow cytometry; IF, immunofluorescence microscopy; SR, spatial RNAseq; E, ELISA; NS, nanostring ncounter; MA, microarray; CY, CyTOF; CS, CITEseq; IHC, immunohistochemistry; GA, Granzyme activity assay; RT, RT-qPCR; MSD, Meso Scale Discovery; IMC, imaging mass cytometry; ICI, immune checkpoint inhibitor; AS, ankylosing spondylitis; SuS, Susac syndrome; MS, multiple sclerosis.

**Figure 2:**
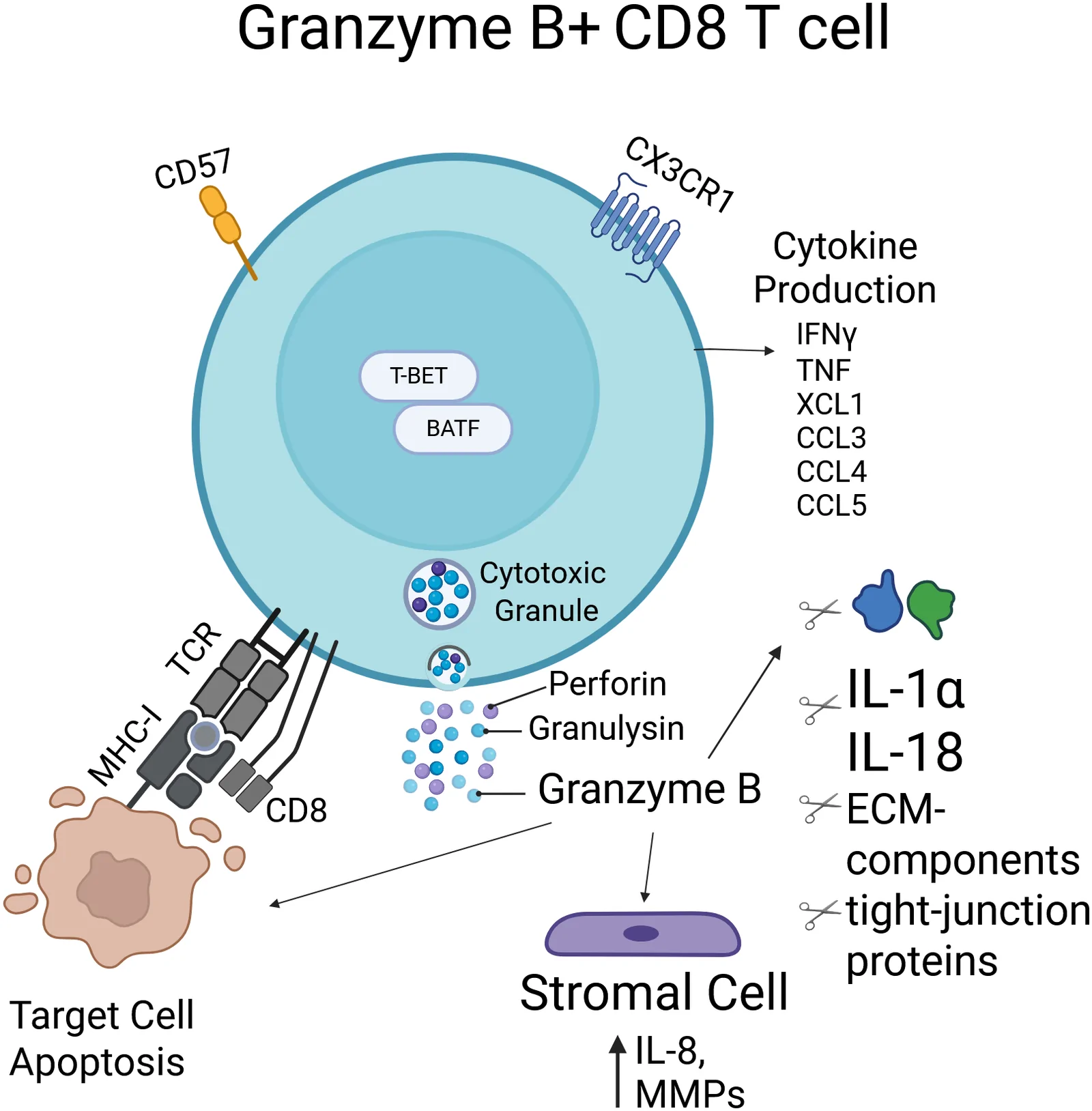
Granzyme B (GZMB) CD8 T cell markers and effector functions.

GZMB can cleave proteins that are not directly related to cytotoxicity as well (**Figure 2**). For example, GZMB cleaves components of tight junctions and extracellular matrix (ECM), including decorin, fibronectin, Von Willebrand factor, plasminogen, fibrinogen, vitronectin, and laminin (108–113). Cleaving these proteins opens up the ECM to allow cells and molecules to infiltrate the tissue (114, 115). Cleavage of ECM also expands cytokine and chemokine gradients since many ECM proteins are negatively charged and bind to positively charged cytokines (116–118). In addition to the release of cytokines from the ECM, GZMB can cleave cytokines IL-1α and IL-18 from their inactive pro form into active cytokines (119, 120).

Beyond GZMB and other cytotoxic granule contents, cytotoxic CD8 T cells produce many other effector molecules (**Figure 2**). The most prominent of these is IFNγ, which induces expression of a number of defensive anti-viral genes and proteins (121). Some of these genes are involved in antigen processing and antigen presentation, resulting in enhanced presentation of peptides to CD8 CTL and thus increased susceptibility to cytotoxic attack if non-self peptides are present (122–124). IFNγ also increases the expression of Fas and TRAIL death receptors as well as multiple caspases, potentiating responses to cytotoxic effectors of CD8 CTL (125–127).

CTLs and other CD8 T cell subsets also produce TNF (tumor necrosis factor) (**Figure 2**). TNF can act on many different cell types. Depending on the context, TNF signaling can induce cell survival, proliferation, transcription of inflammatory cytokines, tissue degradation, and apoptosis (128, 129). CD8 CTL also produce chemokines XCL1, CCL3 (MIP1α), CCL4 (MIP1β), and CCL5 (RANTES), which recruit leukocytes, especially myeloid cells and T cells. These myeloid cells can include macrophage populations and dendritic cells (DC), both type 1 conventional DC and plasmacytoid DCs, which in turn can augment recruitment and activation of additional CD8 T cells and other leukocytes (130).

In single-cell RNA-seq studies, CD8 CTL are generally easy to identify by their robust expression of *GZMB*, *PRF1*, and *GNLY* as well as *CX3CR1* and *XCL1* (**Figure 2**; **Table 1**). In protein-based studies, surface markers such as CX3CR1, CD57, and T_EMRA_ markers (i.e., CD45RA+ CCR7-) are preferentially expressed by CD8 CTL, which are also readily identified by intracellular staining for GZMB protein (usually in the absence of GZMK) (1, 28, 29). As discussed in the next section, not all granzymes are cytotoxic, so expression of granzymes other than GZMB (e.g., GZMA or GZMK) should not be used to identify CD8 CTL.

### Granzyme K+ CD8 T cells

Recent literature has demonstrated that many CD8 T cells in blood and especially tissue have a different phenotype than GZMB+ CTL. These CD8 T cells express GZMK instead of, or in addition to, GZMB and perforin (which, when present, are expressed at lower levels than in CD8 CTL) (**Figure 3**) (1, 131). In many inflammatory conditions, GZMK is expressed by the majority of CD8 T cells present within the tissue (**Table 2**) (1, 132).

**Figure 3:**
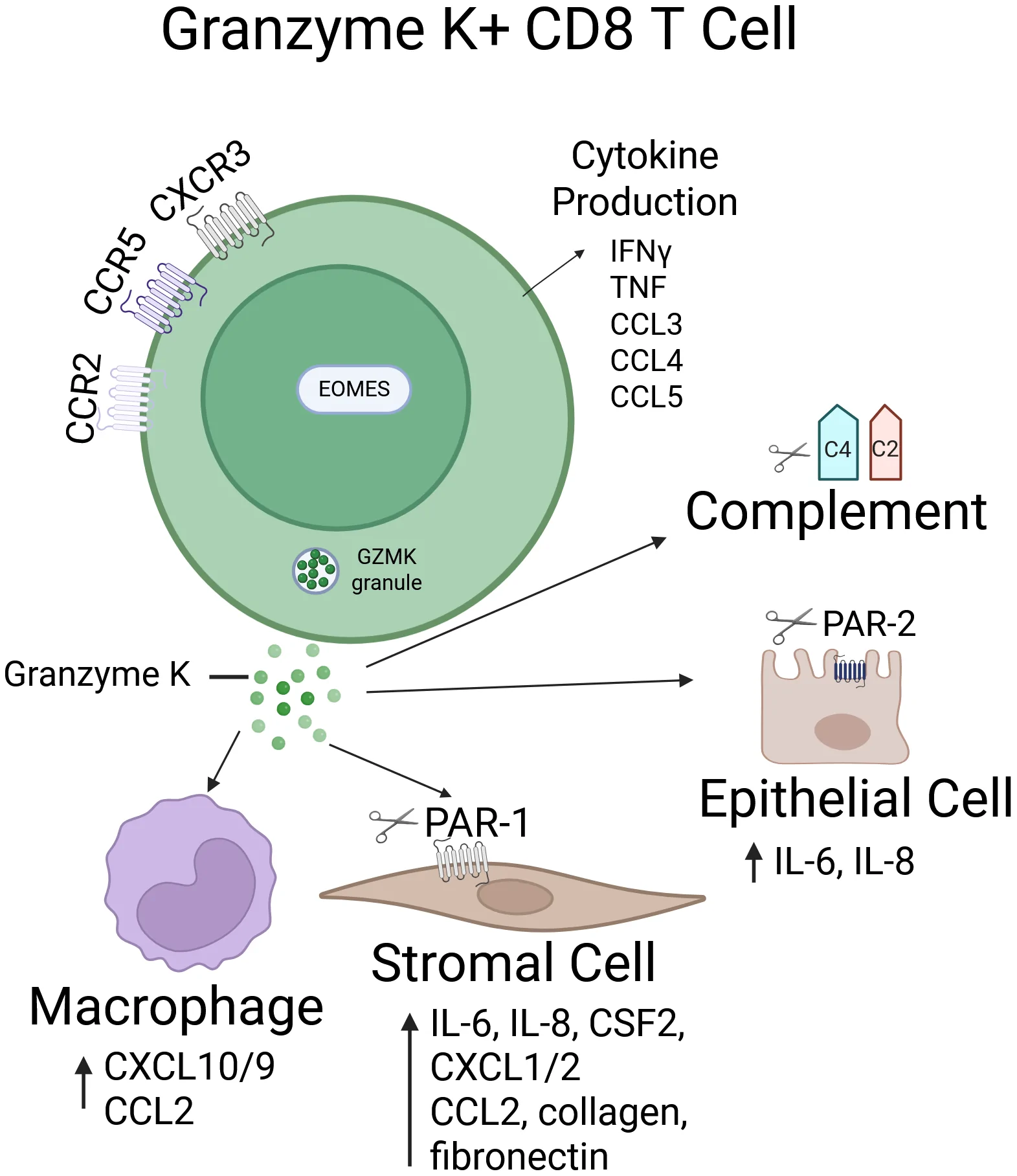
Granzyme K (GZMK) CD8 T cell markers and effector functions.

**Table 2:** GZMK+ CD8 T cells.

Condition	Tissue	Detected as	Method	Notes	Ref.
Aging Related					
Aging	blood	transcript, protein	SC, FC, CY, CS	GZMK CD8 T cells accumulate with age	(244)
Atherosclerosis #	atherosclerotic plaques, blood	transcript, protein	SC, CY, CS, FC	GZMK CD8 T cells enriched in plaques over blood	(249–251)
Ischemic Stroke #	blood	transcript, protein	SC, FC	GZMK CD8 T cell frequency correlates with stroke severity	(255)
Alzheimer’s #*	Cerebrospinal fluid, blood	transcript	SC	GZMK CD8 T cells increase in frequency in CSF and in blood of people with Alzheimer’s compared to controls	(246–248)
Autoimmune					
Rheumatoid Arthritis	synovial tissue/fluid, blood	transcript, protein	SC, B, FC, IF	Most synovial CD8 T cells express GZMK and these cells exhibit moderate, clonal expansion in synovial tissues	(1, 21)
Crohn’s Disease	small intestine	transcript	SC	Higher % of CD8 T cells express GZMK than GZMB	(1, 329)
Ulcerative Colitis	large intestine	transcript	SC	Higher % of CD8 T cells express GZMK than GZMB	(1, 257)
Systemic Lupus Erythematosus	kidney, skin	transcript	SC	Present in both skin and kidney	(133, 243, 330)
Sjogren’s Disease #	salivary glands	transcript, protein	SC, SR, IF, FC	GZMK+ CD8 T cells accumulate with disease severity, GZMK CD8 T cells are enriched in salivary glands, GZMK CD8 colocalize with ducts and acini	(149, 261, 331)
Psoriatic Arthritis	synovial tissue/fluid, serum	transcript, protein	SC, E	GMZK CD8 T cells enriched in synovium over skin	(202, 332)
Hashimoto’s Thyroiditis	thyroid	transcript	SC	Higher % of CD8 T cells express GZMK than GZMB	(133)
Granulomatous Uveitis	Eye	transcript	SC	Majority of ocular fluid CD8 T cells express GZMK with reduced GZMB, perforin, granulysin gene expression	(333)
IgG4-Related Disease	salivary gland	transcript, protein	SC, IF	GZMK+ CD8 T cells enriched in salivary gland and higher frequency than Sjogren’s disease	(260)
Multiple Sclerosis	brain tissue, CSF, bone marrow	protein	FC	GZMK+ CD8 T cells are present in brain tissue, CSF, and bone marrow	(242, 334, 335)
Juvenile Idiopathic Arthritis	synovial tissue	transcript	SR	GZMK+ CD8 T cells enriched in JIA	(336)
Cancer					
Acute myeloid leukemia *#	bone marrow, serum	transcript, protein	SC, FC	GZMK+ CD8 T cells associate with inflammatory phenotype with poorer prognosis, correlate with improved overall survival with PD-1 blockade therapy	(285, 286)
Glioblastoma	brain tumor	transcript, protein	SR, FC	GZMK+ CD8 T cells are enriched among tumor infiltrating lymphocytes compared with matched blood, as both single positive and double positive populations (GZMK+ GZMB+)	(267)
Esophageal Squamous-Cell Carcinoma	esophageal tumor	transcript, protein	SC, IF	GZMK+ CD8 T cells among tumor infiltrating lymphocytes expand more after PD-1 blockade therapy in treatment responders over non-responders	(274)
Intrahepatic Cholangiocarcinoma *	liver tumor	transcript, protein	SC, FC	Increase in GZMK+ CD8 T cells in tumors predicts favorable treatment response and correlates with tumor shrinkage; high GZMK expression level associates with better survival	(271)
Non-Small Cell Lung Cancer	blood, lung tumor, adjacent normal lung	transcript, protein	SC, FC	GZMK+ CD8 T cells expand in response to ICI therapy, subtype with best survival have higher intratumoral GZMK+ CD8 T cells	(272, 337–340)
Breast Cancer *	breast cancer tissues	transcript, protein	NS, IF	Higher expression of GZMK is associated with improved overall survival, progression-free survival, and recurrence-free survival, as well as reduced residual cancer burden and better treatment response to neo-adjuvant chemotherapy and immunotherapy	(276, 277)
Colorectal Cancer *#	colorectal tumors and normal adjacent tissue	transcript, protein	SC, IF	High GZMK+ CD8 T cell tumor infiltration associates with lower survival and early relapse in one study but GZMK+ CD8 T cells are enriched in patients with good outcomes in another	(281, 283)
Clear Cell Renal Cell Carcinoma *	kidney tumor and adjacent tissue	transcript	SC	CD8 T cells clonally expanded after ICI therapy in responders express GZMK and GZMB; GZMK expression correlates with CD8 T cell expansion in responders but not non-responders	(280)
Melanoma *	skin, lymph node	transcript	MA, B	GZMK expression associated with prolonged survival (though not specifically from CD8s), GZMK also associates with overall survival and progression free survival post ICI treatment (though not specifically from CD8s)	(278, 279)
Multiple Myeloma *#	bone marrow	transcript, protein	SC, CY	Rapid progressors are enriched in GZMK+ CD8 T cells compared to non-progressors in one study but, GZMK-expressing CD8 T cells are associated with longer progression free survival after treatment in another	(269, 270)
Follicular Lymphoma #	tumor tissue	transcript	NS	Multiple genes associated with CD8 T cells including GZMK are downregulated in tumors specimens of progressors compared to non-disease progressors	(268)
Lung Adenocarcinoma	lung tumor	transcript	SC	Higher GZMK is associated with favorable ICI therapy response in three different cohorts and higher overall survival, with the exception of a high T_EX_ subgroup	(273)
Infectious Disease					
Tuberculosis	lung tissue, pleural fluid, blood	transcript, protein	SC, FC	GZMK CD8 T cells are enriched in pleural fluid of TPE patients and GZMK protein levels are higher in pleural fluid from patients with TB over those with non-TB pleural effusions	(263–265, 341)
Human Immunodeficiency Virus	blood	transcript, protein	SC, FC	Higher GZMK CD8 T cell frequency correlates with worse clinical markers for HIV, 50% of HIV-1 tetramer antigen specific CD8 T cells express GZMK	(216, 218, 262)
Cytomegalovirus	blood, plasma	protein	FC	In healthy participants with latent CMV, 40-50% of CD8s reactive to HCMV tetramer antigens express GZMK, GZMK is elevated in plasma of those with active CMV infection	(39, 218, 342)
Dengue	blood, plasma	protein	FC	GZMK expression in CD8 T cells more frequent in subclinical infections than hospitalized cases, GZMK is elevated in plasma of those with active Dengue fever	(342, 343)
Influenza A Virus	blood	protein	FC	Most influenza A-specific CD8 T cells express GZMK	(39, 218, 266)
Severe SARS-CoV-2	bronchiolar lavage fluid, blood	transcript	SC	Present in bronchiolar lavage fluid and enriched in PBMC of those with severe COVID-19	(1, 344)
Epstein-Barr Virus	blood	protein	FC	In healthy participants with latent EBV, most EBV-specific CD8 T cells express GMZK	(39, 218)
Hepatitis B	liver, blood	transcript	SC	GZMK CD8 T cells in liver correlate with higher histological grade and higher HBV DNA levels	(345)
Other Conditions					
Chronic Rhinosinusitis with Polyps #	lung, polyps	transcript, protein	SC, FC, IF	GMZK expression enriched in tissue over healthy controls, CD8 GZMK T cells correlate with severe disease	(133, 203)
Eosinophilic Esophagitis	esophagus	transcript	SC	Higher % of CD8 T cells express GZMK than GZMB in both active disease and remission	(133)
Atopic Dermatitis	skin	transcript	SC	Higher % of CD8 T cells express GZMK than GZMB	(133)

*Favorable or #unfavorable prognostic associations with GZMK CD8 T cells; ICI, immune checkpoint inhibitor; CSF-cerebrospinal fluid; SC, scRNAseq; B, bulk RNAseq; FC, flow cytometry; IF, immunofluorescence microscopy; SR, spatial RNAseq; E, ELISA; NS, nanostring ncounter; MA, microarray; CY, CyTOF; CS, CITEseq.

GZMK+ CD8 T cells have several pro-inflammatory effector functions in tissues, including release of cytokines, chemokines, and GZMK, in response to TCR-mediated or cytokine-mediated stimulation (1, 133) (**Figure 3**). These factors in turn exert numerous effects on surrounding cells. As discussed for CD8 CTL above, IFNγ activates antiviral responses and promotes antigen processing and presentation by antigen-presenting cells, among other effects (61, 121, 124). TNF has pro-inflammatory effects on a broad array of cells, including stromal cells (128, 129). IL-2 is a survival and proliferative factor for T cells (134). GZMK+ CD8 T cells can also express CCL3, CCL4, and CCL5, which can recruit DCs, myeloid cells, and T cells (1, 21). Of note, unlike CD8 CTL, GZMK+ CD8 T cells do not typically express XCL1 (1).

GZMK+ CD8 T cells also secrete GZMK, a protease with different cleavage specificities than GZMB (135–137). Instead of activating apoptotic caspase cascades, GZMK has several non-cytotoxic proinflammatory effects on target cells, including activation of complement cascades, expression of cytokines and chemokines by fibroblasts, macrophages, endothelial cells, and epithelial cells, and increasing expression of fibrosis proteins such as collagen type 1 and fibronectin by fibroblasts (1, 137–140) (**Figure 3**). These effects are mediated, at least in part, by cleavage of protease activated receptors (PARs), a family of G-protein-coupled receptors (GPCRs) expressed on the surface of many cell types (141). GZMK-mediated cleavage of PAR-1 is the most well-studied of these interactions. Cleavage of PAR-1 on endothelial cells, keratinocytes, or fibroblasts results in increased cytokine production of proinflammatory cytokines including IL-6, IL-8, IL-1β, and CCL2 (138, 142–144). GZMK-induced PAR-1 cleavage also results in increased adhesion molecule expression (ICAM1, VCAM-1, E-selectin) in endothelial cells (138). GZMK can cleave PAR-2, which in epithelial cells results in release of IL-6 and IL-8 (139). GZMK can also cleave PAR-4, though the downstream effects of GZMK cleavage of PAR-4 have not yet been determined (145). GZMK interactions with lipopolysaccharide (LPS) (of gram-negative bacteria) lead to enhanced interactions with CD14 and therefore potentiates TLR4 proinflammatory signaling (146). Lastly, GZMK can directly cleave complement proteins C2 and C4 into C4b and C2b, leading to complement cascade activation (**Figure 3**) (137). Complement cascades have many downstream effector functions, including production of anaphylatoxins C3a and C5a, deposition of opsonin C3b on nearby antigens and cell surfaces, and formation of the membrane attack complex (MAC) (137). C3a and especially C5a are potent chemoattractants and stimulants of granulocytes and myeloid cells, while C3b induces B cell activation and differentiation as well as antigen uptake by APCs (147).

The mechanisms regulating GZMK expression and release are not fully understood. TCR stimulation leads to an immediate decrease in intracellular GZMK protein in CD8 T cells while cytokine stimulation with IL-2/IL-12 or IL-15 induces moderate GZMK expression (137, 148, 149). In CD4 T cells, transcription factor EOMES induces GZMK expression (150, 151), though the role of EOMES in GZMK expression by CD8 T cells has not been determined. The mechanisms driving the accumulation of large numbers of GZMK+ CD8 T cells in inflamed tissues and triggering their release of GZMK warrant further investigation.

### Exhausted CD8 T Cells

T cell exhaustion is a cell phenotype that progressively occurs in response to persistent antigen exposure and results in decreased functional capacity and proliferation. Exhausted CD8 T cells (T_EX_) express inhibitory receptors such as PD-1, and they can be further subclassified as T progenitor exhausted (T_PEX_), T effector-like intermediate exhausted(T_EX_^INT^), and terminally differentiated exhausted cells (T_EX_^TERM^) (**Figure 4**) (152). Transcription factors NFAT, TOX, and NR4A1 are central to T_EX_ development, and environmental factors such as metabolites can also play a role in regulating T_EX_ fate (16, 153–156). Cytokines such as TGFβ, IFNα/β, IL-6, TNF, IL-27 IL-35 can further drive exhaustion (157). T_EX_ cells lose their effector function in a progressive fashion during chronic antigen stimulation. Early changes include loss of IL-2 production, then lower TNF, and lastly reduced IFNγ production (158, 159). T_EX_ cells also lose proliferative and cytotoxic capabilities (157). Importantly, while their effector activity is diminished as terminally exhausted cells, T_EX_ cells are not fully inert and retain some capacity to affect immune responses (160–164). For example, exhausted cells can continue the production of chemokines CCL3, CCL4, and CCL5, as well as the inflammation-dampening cytokine IL-10 (164, 165).

**Figure 4:**
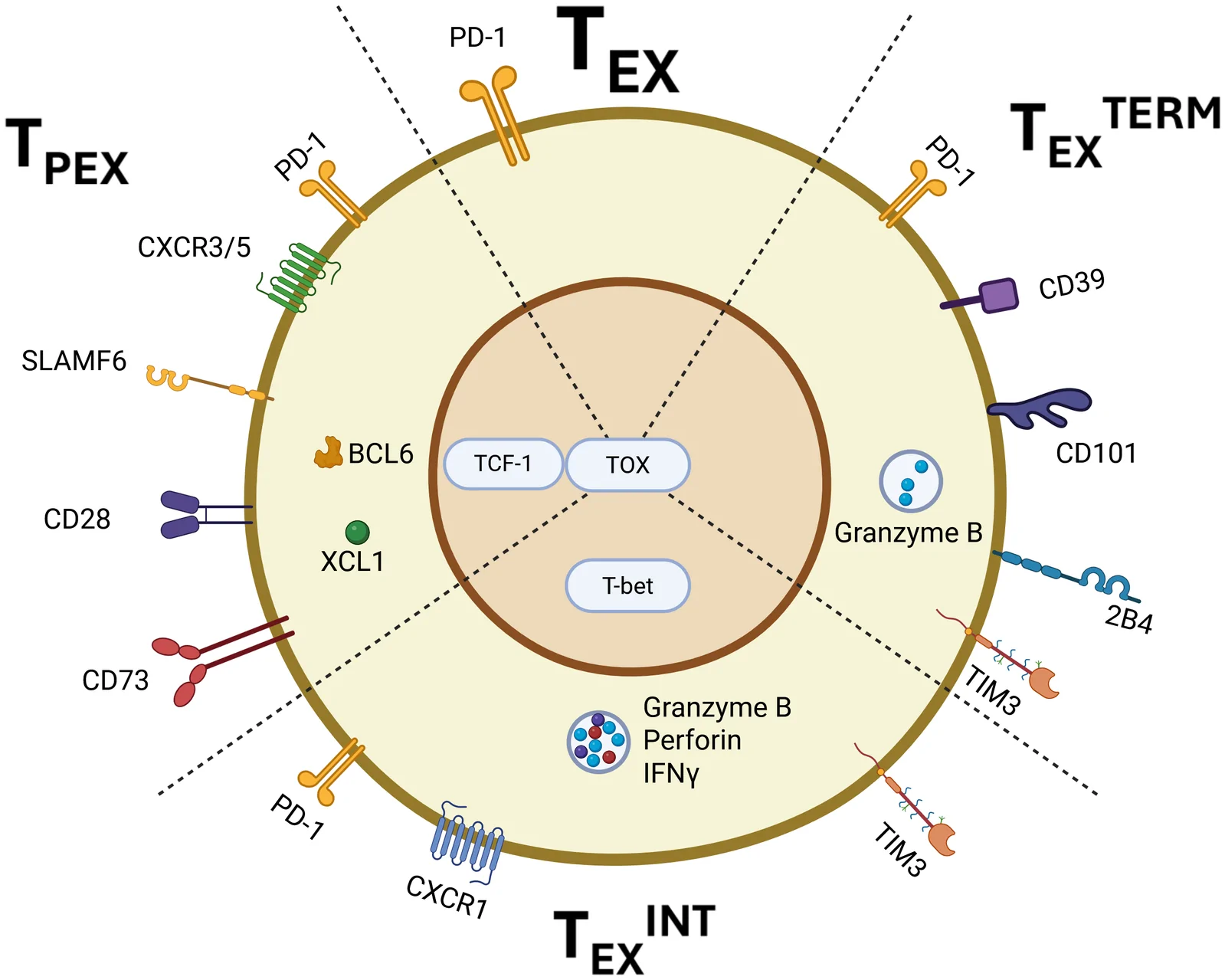
Exhausted CD8 T cell (T_EX_) markers in each class. T_EX_ all exhausted CD8 T cells. T_PEX_ T progenitor exhausted cells. T_EX_^INT^ T intermediate exhausted cells. T_EX_^TERM^ terminally exhausted CD8 T cells. PD-1 and TOX are expressed by all three subsets and are core elements of T_EX_ cells in general.

T_PEX_ cells are also called stem-like progenitor CD8 T cells (not to be confused with stem cell memory (T_SCM_) CD8 T cells). These cells have reduced potential for effector functions but also have some characteristics of stem cells, such as self-renewal. T_PEX_ cells have the ability to differentiate into T_EX_^INT^ cells, and T_EX_^TERM^ T cells, but they can also differentiate into fully potent effector cells(**Figure 4**) (166–168). T_PEX_ cells have distinct markers compared to T_EX_^INT^ and T_EX_^TERM^ including SLAMF6, CD28, and CD73 expression(**Figure 4**) (30).TCF-1 drives the generation of T_PEX_ cells, which are also regulated by TOX and BACH2 (169–171). T_PEX_ cells are mainly located in secondary and tertiary lymphoid tissues, but they can also migrate to tissue and cause damage (30). In systemic lupus erythematosus, T_PEX_ cells differentiate into effector CD8 T cells upon entrance into the kidney, acquire cytotoxic effector functions, proliferate, and cause kidney damage (172). Some T_PEX_ CD8 T cells express GZMK+, and are further characterized by expression of PD-1, TIGIT, and other inhibitory receptors (173). Additional studies are needed to refine the markers that distinguish GZMK+ T_PEX_ cells from other GZMK+ CD8 T cells.

T_EX_^INT^ or effector-like exhausted CD8 T cells (T_EX_^EFF^) are transitional exhausted cells. Proliferating T_PEX_ may develop into T_EX_^INT^ that exhibit cytotoxic effector functions similar to CD8 CTL, including GZMB- and perforin-mediated apoptosis (166, 174). While T_PEX_ cells tend to remain in lymphoid tissues, T_EX_^INT^ cells can migrate to sites of cancer or infection (30, 174).

Terminally exhausted CD8 T cells (T_EX_^TERM^) exhibit further loss of effector functions, impaired proliferative capacity and an inability to respond to stimulation or immune checkpoint blockade (157, 160, 175). T_EX_^TERM^ have altered metabolism, specifically impaired glycolysis and oxidative phosphorylation (176). Unlike T_PEX_ and T_EX_^INT^ cells, T_EX_^TERM^ cannot revert to an effector phenotype.

Overall, T cell exhaustion likely evolved as a protective mechanism to avoid acute immunopathology that can result from unrestrained CD8 T cell activation. However, T cell exhaustion also results in reduced effectiveness of immune responses against chronic infections and cancer (177). Especially in the oncology field, many efforts have been made to prevent or reverse T cell exhaustion with the goal of enhancing anti-tumor responses. Immune checkpoint inhibition, consisting of antibodies blocking PD-1 or other inhibitory receptors, is a highly successful example of this. These treatments primarily target T_PEX_ and T_EX_^INT^ cells, inducing them to expand in number, upregulate cytotoxic effector factors, and attack tumor cells (178–181).

Early gene signatures used to identify T_EX_ and T_RM_ CD8 T cells have a large amount of overlap and, for many years, these cell subsets could not be reliably differentiated (10, 11). Furthermore, surface protein expression of inhibitory receptors such as PD-1 can indicate recent TCR-mediated stimulation, instead of exhaustion (10). Recent work has shown that T_RM_ and T_EX_ are clonally distinct, and specific gene signatures have been created within the context of some diseases to separate these cell types more effectively, but care must be taken when interpreting older literature (10, 11). There is also some ambiguity between GZMK+ CD8 T cells and T_EX_ cells. T_EX_, and in particular T_PEX_ cells, can express GZMK, and they also exhibit progressive loss of cytotoxic effector functions, often measured by protein or gene expression of GZMB and perforin (155, 157). GZMK+ CD8 T cells similarly have lower GZMB and perforin yet, as a population, have intact effector function and thus do not all represent T_EX_ cells (1, 133). For these reasons, T_PEX_ cells and GZMK+ CD8 T cells should not be conflated based on GZMK expression alone.

### Regulatory CD8 T cells

Regulatory CD8 T cells (CD8 Treg) are a heterogenous subset of CD8 T cells that express some classical CD4 Treg proteins and have potential immunosuppressive function. Due to low CD8 Treg frequency *in vivo* and inconsistent phenotypic markers, this cell subset is not as well-studied as their CD4 Treg counterparts (182). However, protective CD8 Treg suppressive effects have been described in multiple disease contexts including systemic lupus erythematosus (SLE), rheumatoid arthritis (RA), multiple sclerosis (MS), inflammatory bowel disease (IBD), and graft versus host disease (GVHD), as recently reviewed (15, 182). CD8 Tregs have been described as capable of suppressing immune responses by killing autoreactive CD4 T cells (15, 183, 184) and apoptosis through FAS signaling (185). In addition to direct cytotoxicity, CD8 Tregs can suppress immune function through production of suppressive cytokines such as IL-10 and TGFβ (186), negative regulation through CTLA-4 or PD-1 (187, 188), and blocking T cell proliferation by competing for IL-2 with CD25 expression (189, 190).

Treg CD8 T cells express FOXP3, IL-10, TGFβ, IL-34, IL-35, CX3CR1, and Helios, similar to CD4+ Tregs (14, 191). Other markers reported to differentiate Treg CD8s from other CD8 T cells include CD25, CD122, CD103, CTLA-4, CD39, LAG-3, and KIRs (killer cell immunoglobulin like receptor) in humans and Ly49 in mice (15, 192). CD8 T regs generally lack CD28, CD127, and CD49d (15). KIR+ CD8s can target and kill autoreactive CD4 T cells through HLA and HLA-E recognition (193). In tissues specifically, CD8 Tregs could contribute to the balance between effective response and tissue damage from the immune response (182). This role in balancing immune responses is exploited by some pathogens and cancers. *Mycobacterium* species have been observed to induce the suppressive activity of CD8 Tregs as an immune evasion tactic to survive (191, 194, 195). CD8 Tregs have also been found in breast, ovarian, colorectal, and prostate cancers, where they are thought to impede anti-tumor responses through multiple mechanisms, including TGFβ and IL-10 secretion (196–199).

### Anergic CD8 T cells

Anergic CD8 T cells are hyporesponsive cells that do not produce IL-2 or proliferate upon antigen re-encounter. Anergic cells develop when a naïve CD8 T cell receives primary MHC-I antigen stimulation but does not receive co-stimulatory signals through CD28, CD80, or other co-stimulatory receptors (200). In other words, anergic CD8 T cells are functionally impaired similar to T_EX_ cells but develop through distinct mechanisms, namely the lack of co-stimulation during initial TCR stimulation as opposed to prolonged TCR-mediated stimulation. In mice there are accepted markers of anergic T cells (CD44+ FR4+ CD73+ FOXP3- CD304+ CXCR5-) (30). In contrast, markers of human anergic T cells are not well defined, a major obstacle to their study (30).

### Two major CD8 T cell subsets defined by functional molecules

The complexity of CD8 T cell subsets represents a challenge to the translational immunology research community. Many of the subsets overlap with each other or are difficult to distinguish without functional assays, which are difficult to perform in human samples (e.g., assessing whether a CD69+ CD8 T cell in a tissue biopsy specimen is a true T_RM_ cell or an activated CD8 T cell transiently present in the tissue). As such, a framework that relies on a limited number of markers to classify CD8 T cells by effector function would be useful, especially if the markers are easy to detect at both the mRNA and protein levels. We propose that GZMK and GZMB provide this framework.

The relevance and utility of classifying CD8 T cells based on GZMK and GZMB is supported by several important observations. First, GZMK and GZMB expression correlates well with the classic CD45RA/CD45RO/CCR7 classification system for blood cells (28, 29). Naïve cells (defined by surface markers CCR7 and CD45RA) express neither GZMB nor GZMK. CD8 T cells that receive strong T cell stimulation strongly upregulate GZMB and downregulate GZMK, consistent with differentiation into CD8 CTL (137). T_CM_ and T_EM_ CD8 T cells mainly express GZMK alone while T_EMRA_ cells mainly express GZMB alone (1, 28, 29). A small percentage of circulating CD8 T cells and a larger portion of CD8 T cells in tissues, express GZMB and GZMK together (1).

Second, GZMK and GZMB at the mRNA and protein level are convenient markers of distinct larger transcriptional programs that define the two dominant effector phenotypes of CD8 T cells, namely a GZMB-associated cytotoxic effector function program versus a GZMK-associated non-cytotoxic, pro-inflammatory effector function program (1, 21). For researchers analyzing -omics datasets from human diseased tissues, correctly identifying the effector function phenotype of CD8 T cells is crucial to understanding their role in disease.

Third, GZMK+ and GZMB+ CD8 T cells are largely clonally distinct in humans *in vivo (*1, 201, 202). Sister clones within a given clonotype (i.e. CD8 T cells with identical TCR sequences that likely originate from the same initial activated naïve CD8 T cell) tend to have the same phenotype, i.e., most of the cells in a clonotype express either GZMB alone or GZMK with or without GZMB (1, 133, 149, 201–203). This consistent phenotype of CD8 T cells within a clonotype holds true even across tissue compartments – sister clones in blood and synovial tissue exhibit similar transcriptomic profiles, i.e., sister clones from a given clonotype belong to a GZMK+ cluster regardless of whether they came from blood or tissue (1, 201, 202). These findings indicate that CD8 T cells do not commonly switch from one subset to the other, indicating in turn that these phenotypes are long-lived *in vivo*, adding durability and cross-tissue relevance to this classification system. GZMK and GZMB are therefore useful markers of two distinct populations of CD8 T cells that do not intermix.

This is not to say that other CD8 T cell subsets are not relevant. For example, the identification and study of T_RM_ CD8 T cells has revealed important intrinsic CD8 T cell biology, and a large population of CD8 T cells expressing T_RM_ markers such as ZNF683 and CD103 in a given tissue suggests that the CD8 T cell response in that tissue is not very migratory. In other situations, researchers may choose to focus on identifying cell subsets that are important for predicting treatment targets or responses, such as identifying exhausted CD8 T cell subsets within tumor microenvironments. However, designating cells as CD8 T_RM_ or T_EX_ T cells tells us mainly about their migratory potential or their likelihood to resume potent effector functions after checkpoint blockade. These designations do not tell us much about their effector function of information when trying to understand the role of CD8 T cell subsets in the pathogenesis of a particular disease. The GZMB versus GZMK classification system fills this gap, especially when deep characterization of CD8 T cells is not a major goal of the study. Examining GZMK and GZMB expression as a simple surrogate of GZMK- vs GZMB-associated transcriptional programs (or the full transcriptional programs in the case of RNA-seq data) can provide rapid insight into the potential targetable effector functions exercised by those CD8 T cells.

In the sections below, we review CD8 T cell findings from human tissues through the lens of *GZMK* and *GZMB* expression. By understanding the pattern of enrichment of these two CD8 T cell subsets and, when available, their association with disease prognosis or treatment response, we can get a better sense of their roles in disease pathogenesis across a range of human diseases.

## Disease associations of GZMB+ CD8 T cells

GZMB+ CD8 CTLs are found in blood and many tissues in healthy humans including the lungs, intestine, skin, liver, female reproductive tract, kidney, lymph nodes, brain, heart, pancreas, and salivary glands (92, 204, 205). As discussed for GZMB+ CD8 CTLs above, the main recognized role for GZMB+ CD8 T cells in tissue is to kill infected or malignantly transformed cells (**Figure 2**), and accordingly, their presence in tissues are protective in many infections and cancers but are harmful in certain autoimmune conditions (**Table 1**).

### GZMB+ CD8 T cells in infections

GZMB+ CD8 T cells are associated with a favorable prognosis in many viral infections (**Table 1**). For example, GZMB+ CD8 CTL correlate with protection against severe influenza (206–209). Increased cytotoxic activity of CD8 T cells correlates with faster recovery, reduced symptoms, and fewer fatalities from H1N1 or H7N9 influenza A virus infections (210, 211). Respiratory syncytial virus (RSV)-specific CD8 T cells post-infection are enriched in the lung over the blood (212) and express more granzyme B after viral challenge (213, 214). In mouse studies of Zika virus (ZIKV), adoptive transfer of ZIKV-challenged CD8 T cells are protective against neuroinflammation and exhibit higher GZMB expression (215). In people with HIV-1, GZMB+ CD8 T cells are more frequent in both blood and colon tissue compared to people without HIV-1 (216, 217). The majority of HIV-1-specific CD8 T cells reactive to viral peptides express GZMB (39, 218). During HIV-1 infection, CD8 T cells specific for other viruses, such as HCMV, also more frequently express GZMB (39). HIV-1-specific CD8 T cells from elite controllers with HIV-1 (i.e., the approximately 1% of people who maintain long-term viral suppression without antiviral medication) express GZMB and perforin at a higher frequency than CD8 T cells from progressors (219, 220).

### GZMB+ CD8 T cells in malignancy and cancer immunotherapies

Protective effects of GZMB+ CD8 T cells extend to cancer as well, including colorectal cancer, lung cancer, breast cancer, gastric cancer and others (**Table 1**). In colorectal cancer, CD8 T cell presence and GZMB protein expression in tumor tissue associates with survival (221, 222). Interestingly, expression of SerpinB9, an endogenous GZMB inhibitor, in colorectal cancer cells associates with lymph node metastasis (222). In triple-negative breast cancer, high frequency of CD8 T cells expressing GZMB in tumor tissue correlates with higher overall survival and disease-free survival (223). A higher frequency of GZMB+ CD8 T cells among tumor infiltrating lymphocytes associates with higher overall survival in gastric cancer (224), and multiple forms of non-small cell lung cancer have better prognosis with GZMB+ CD8 T cells (225–228). Expression of GZMB is a positive prognostic marker for overall survival and disease-free survival in invasive breast carcinoma, lung adenocarcinoma, cutaneous melanoma, and hepatocellular carcinoma (229, 230).

The presence and activity of CD8 CTL is important for the response to immunotherapy in many cancers, as recently reviewed (231, 232). The main immunotherapies effecting CD8 T cell function in cancer treatment include immune checkpoint inhibitors (ICI), cancer vaccines, and cell therapies such as chimeric antigen receptor T (CAR-T) cells (231). ICI therapies are antibodies that block inhibitor receptors and their ligands, such as PD-1, PD-L1, and CTLA-4, and they have been approved to treat melanoma, colon cancer, Hodgkin lymphoma, liver, kidney, and lung cancer among others (233). These treatments release T_EX_^INT^ and T_PEX_ CD8 T cells from tonic inhibitory signaling, allow them to upregulate GZMB and execute cytotoxic effector functions toward cancer cells (17). CAR-T cells are CD8 T cells that have been programmed to express an engineered activating receptor targeting a specific antigen, such as CD19 on B cells of B-cell lymphoma (234). The main killing mechanism of CAR-T cells is GZMB-induced apoptosis (234).

### GZMB+ CD8 T cells in autoimmunity and solid organ transplantation

Despite their beneficial role in suppression and control of many infections and cancers, there are some diseases in which CD8 CTL have negative effects (**Table 1**). Several of these are autoimmune in nature and feature CD8 T cell-mediated destruction of tissues, such as β-cells in type 1 diabetes and melanocytes in vitiligo (80, 235, 236). CD8 CTL may also contribute to detrimental inflammation in other ways, such as production of cytokines and chemokines that add to the inflammatory environment. GZMB+ CD8 T cells also have detrimental effects on post-transplantation rejection in multiple tissues including the heart, kidney, liver, and lung (237–241). For example, liver tissues during rejection have more CD8 T cells, and the CD8 T cells present upregulate GZMB expression (241).

## Disease associations of GZMK+ CD8 T cells

In healthy humans, GZMK+ CD8 T cells have been observed in many tissues, including skin, liver, adipose, kidney, synovial tissue, and bone marrow, in addition to bronchiolar lavage fluid (1, 148, 242, 243). Beyond healthy tissues, GZMK+ CD8 T cells are present and enriched in multiple autoimmune conditions, cancers, and infectious diseases (**Table 2**). GZMK has long been known as a gene expressed by T_CM_ and T_EM_ CD8 T cells in blood in healthy individuals (28, 29), but recent single-cell RNA-seq studies have revealed that this cell population is more abundant and more complex than originally thought. Our understanding of their role in disease pathogenesis remains incomplete (**Figure 3**). Here, we present a review of the current literature.

### GMZK CD8 T cells in aging and aging-related conditions

GZMK+ CD8 T cells accumulate in blood and tissues with age and have been connected to inflammaging (**Table 2**) (244). Their exact role in that process remains unclear. Even within a given disease class, such as dementia, some reports suggest that GZMK+ CD8 T cells contribute to disease, while others indicate that GZMK+ CD8 T cells can help protect against disease (245, 246). In people with Alzheimer’s disease, there is a significant increase in the frequency of GZMK+ CD8 T cells over healthy controls in both the PBMC and cerebrospinal fluid (CSF), and in CSF, GZMK+ CD8 T cells increase with disease severity (247, 248). Recent studies have also detected GZMK+ CD8 T cells in brain tissue of mouse models of Alzheimer’s disease, though these studies disagree on the role of GZMK in disease pathogenesis. In one study, the presence of GZMK+ CD8 T cells is beneficial for clearance of tau-rich microglia, slowing disease progression (246), while the other study found that GZMK is harmful in Alzheimer’s disease by increasing neurotoxic inflammation through PAR-1 signaling leading to hyperphosphorylation of tau in neurons (248).

The presence of GZMK+ CD8 T cells is considered to be detrimental in other conditions in which age is a significant risk factor, including atherosclerotic plaques and ischemic stroke (**Table 2**). In atherosclerotic plaques, T cells are the most abundant immune cell type, with CD8 T cells being the most highly enriched compared to the blood (249, 250). The vast majority of CD8 T cells in plaques express GZMK, which is one of the top differentially expressed genes in plaque versus blood CD8 T cells (249, 251). Complement cascades (252) and PAR-1 cleavage (253, 254) are both present in atherosclerosis, potentially reflecting GZMK activity in the plaques. Ischemic stroke brain injury may also be impacted by the presence of GZMK+ CD8 T cells through their contributions to neuroinflammation, since GZMK+ CD8 T cell expansion correlates with severity of stroke symptoms in acute ischemic stroke patient PBMC (255). The drivers that lead to accumulation of GZMK+ CD8 T cells with age and the effects this has on the body warrant further investigation.

### GZMK+ CD8 T cells in autoimmune conditions

GZMK+ CD8 T cells are found at tissue sites of inflammation in many different autoimmune diseases, perhaps reflecting the link between inflammaging and autoimmunity. The topic of GZMK+ CD8 T cells in autoimmune diseases was recently reviewed (132), so what follows is a brief summary of the most prominent findings and updates.

GZMK+ CD8 T cells were initially reported as an enriched tissue population distinct from CD8 CTL in inflamed synovial tissue from patients with RA (1). About 70% of CD8 T cells in synovial tissue and fluid express GZMK, either alone or alongside GZMB, compared with about 30% of CD8 T cells in blood (1). These GZMK+ CD8 T cells exhibit modest clonal expansion in synovial tissue, and cells belonging to these clonotypes are also detected among GZMK+ CD8 T cells in blood, suggesting that these cells are not tissue-resident but instead recirculate (1, 201).

GZMK+ CD8 T cell enrichment among T cells in inflamed tissues is remarkably constant across diseases (**Table 2**). GZMK+ CD8 T cells are also found in synovial tissue in psoriatic arthritis (PsA), another autoimmune arthritis, and in osteoarthritis (OA), a non-autoimmune, degenerative arthritis (1, 202). Even within RA, the cellular composition of synovial tissue can vary significantly between individuals, but GZMK+ CD8 T cells constitute a roughly equal proportion among T cells across individuals (21). One important distinction here is that synovial tissues in RA and PsA contain large numbers of CD8 T cells (and other leukocytes), while OA has a much smaller absolute number of CD8 T cells relative to total cell number, but the proportions of GZMK+ CD8 T cells among those T cells are similar (1, 256). An analogous scenario is seen in IBD, where GZMK+ CD8 T cells outnumber GZMB+ CD8 T cells in both healthy and inflamed tissues in Crohn’s disease and ulcerative colitis, but the absolute number of CD8 T cells is low in healthy tissues and higher in inflamed bowel (1, 257, 258).

There are some differences in frequency between tissues in the same disease that are worth noting. First, GZMK expression is more frequent among CD8 T cells in synovial tissue than in skin among patients with PsA (202). This is in contrast to SLE, where the majority of CD8 T cells in skin biopsies from patients with SLE express GZMK, while an equal frequency of CD8 T cells in kidneys affected by lupus nephritis express GZMK and GZMB (1, 243). These observed differences may link to the pathophysiology of these diseases, but those connections have not yet been elucidated. Whether the effector functions of GZMK+ CD8 T cells are the same in these different tissues is also unknown.

Recent studies have reported GZMK+ CD8 T cells in salivary glands affected by Sjogren’s disease, an autoimmune disease characterized by autoimmune destruction of salivary gland epithelial cells that typically produce saliva (259, 260). In Sjogren’s disease, these cells also express GZMB alongside GZMK, but clonally overlap with GZMK+ CD8 T cells in blood more frequently than GZMB+ CD8 T cells, similar to the GZMK+ CD8 T cells in RA (149). These CD8 T cells are located near acinar epithelial cells in Sjogren’s salivary gland biopsies, and *in vitro* experiments, GZMK+ CD8 T cells induce IFN signaling and other signs of dysfunction in cultured acinar cells (261). GZMK+ CD8 T cells have also been reported at an even higher frequency in salivary gland biopsies from patients with IgG4-related disease (IgG4-RD), a multi-organ disease characterized by an infiltration of IgG4+ B cells (260). Both Sjogren’s disease and IgG4-RD feature salivary gland damage and acinar cell loss, and CD8 T cells are thought to mediate this cytotoxicity. Whether GZMK+ CD8 T cells directly kill the acinar cells, and if so, by which mechanism (e.g., release of concurrently expressed GZMB, Fas/FasL, or cytokine-mediated effects), has not been conclusively determined.

### GZMK+ CD8 T cells in infections

GZMK CD8 T cells have been observed during and after the resolution of several infectious diseases (**Table 2**). People with HIV-1 (PWH) have higher frequency of GZMK+ CD8 T cells in the blood compared to people without HIV-1 (216). This expansion of GZMK CD8 T cells occurs in PWH independent of antiretroviral therapy (ART) and viral control in response to ART (262). A high frequency of GZMK CD8 T cells correlates with worsening clinical markers of disease severity including lower CD4/CD8 ratios, a measurement of viral depletion of CD4 T cells, and high concentration of HIV-1 DNA, suggesting that GZMK CD8 T cells have a negative impact on immune control of HIV-1 (262).

In individuals with active pulmonary tuberculosis (TB) infection, cells present in pleural fluid and lung tissue, are enriched for GZMK+ CD8 T cells (263, 264). Interestingly, GZMK+ CD8 T cells are more enriched among CD8 T cells in pleural effusions due to TB compared to malignant or parapneumonic pleural effusions (264). Unlike in HIV-1, where GZMK CD8 T cell frequency correlates with disease severity, in TB, GZMK CD8 T cells frequency in peripheral blood inversely correlates with the severity of TB lesions in lung tissue (265). The role and mechanistic effect of GZMK CD8 T cells in tuberculosis infections has not yet been determined.

In contrast to HIV-1 and TB, GZMK may have favorable effects during influenza infection. In blood, 20-60% of Influenza A virus-specific CD8 T cells express GZMK without GZMB or perforin (39, 218). In influenza A infection, GZMK+ CD8 T cells may be able to directly block viral replication since GZMK can cleave importin α1 and β *in vitro*, thus preventing nuclear transport of IAV nucleoprotein and impairing IAV replication (266).

### GZMK+ CD8 T cells in cancer

GZMK-expressing CD8 T cells are found in multiple malignancies, including glioblastoma, esophageal carcinoma, follicular lymphoma, multiple myeloma, liver cancer, lung cancer, and pancreatic cancer (**Table 2**) (267–275). Their contribution to immune control or immune support within these cancers is currently unknown and requires further research. In the meantime, there is a growing amount of circumstantial data in the form of associations with favorable or unfavorable outcomes, but these studies do not point in a uniform direction, perhaps because T_EX_ subsets represent a variable portion of the GZMK+ CD8 T cell subsets. Here we summarize a few examples.

Association of GZMK+ CD8 T cells with favorable prognosis or treatment response has been reported for several malignancies including breast cancer, melanoma, renal cell carcinoma (**Table 2**). In breast cancer, high GZMK expression associates with higher overall survival, progression-free survival, and recurrence-free survival, and higher expression of GZMK correlates with responsiveness to treatment (276, 277). In melanoma, GZMK+ CD8 T cells are also associated with favorable prognosis. The expression of GZMK in melanoma lesions post-recurrence correlates with increased survival overall, regardless of treatment (278). Responders also have a higher GZMK expression level compared to non-responders among cancer patients treated with antibodies targeting melanoma tumor antigen MAGE-A3 or ICI therapy targeting PD-1 (276). *GZMK* RNA expression is upregulated in people with clinical response to multiple immune checkpoint inhibitor treatments compared to those that did not respond (279). In clear cell renal carcinoma, co-expression of GZMK and GZMB identified a CD8 T cell subset that would respond to an anti-PD-1 ICI therapy, and individuals with a higher frequency of GZMK+ CD8 T cells had better treatment outcomes after ICI treatment (280).

Other cancers have mixed reported associations for the presence of GZMK+ cells and prognosis (**Table 2**). In colorectal cancer, GZMK+ CD8 T cells are enriched in colon tissue from people who have good outcomes (281). Extracellular GZMK in the context of colorectal cancer has also been shown to upregulate sVEGFR1 on endothelial cells to inhibit angiogenesis of tumors and correlates with decreased tumor size (282). A different study investigating non-metastatic colorectal cancer found an interaction between CD15+ neutrophils inducing GZMK expression in CD8 T cells leading to poorer clinical outcomes (283). However, in another study, CD66b+ neutrophils are associated with increased survival and co-localize with CD8 T cells, contributing to increased activation, proliferation, and cytokine release (284).

Acute myeloid leukemia (AML) also presents mixed associations for GZMK+ CD8 T cell frequency and prognosis. GZMK+ CD8 T cells are the majority of CD8 T cells in bone marrow of responders to immune checkpoint therapy in acute myeloid leukemia, and higher GZMK+ CD8 T cell frequency associates with improved survival (285). Pediatric AML patients with high-inflammation have expanded GZMK CD8 T cells and worse survival outcomes compared to those with lower inflammation (286). However, the GZMK CD8 T cell expansion was not seen in adult patients with high inflammation (286), indicating the contribution of GZMK CD8 T cells to disease progression may be conditional.

### GZMK+ CD8 T cells in allergic and upper respiratory conditions

Recently, studies of CD8 T cells in multiple lung pathologies report that CD8 T cells expressing GZMK play an important role in disease pathogenesis (**Table 2**). In chronic rhinosinusitis with polyps, which effects 2-2.5% of the population, tissue levels of GZMK, but not serum levels of GZMK, are elevated, especially in individuals with severe nasal polyps (203). CD8 T cell subsets expressing GZMK correlate with disease severity (203), possibly by promoting neutrophilic inflammation through interactions with fibroblasts (133). In humans, the abundance of GZMK+ CD8 T cells in lung tissue of people who have nasal polyps resections are predictive of asthma comorbidity (203). In a mouse model of asthma, GZMK deficiency results in fewer eosinophils in bronchioalveolar lavage fluid, lower airway inflammation, and reduced asthma tissue pathology (203). GZMK+ CD8 T cells are also enriched, and higher in frequency than GZMB+ CD8 T cells, in esophagus and duodenum tissue samples of eosinophilic esophagitis (133).

## New discoveries on the horizon

In this section, we discuss some ongoing questions yet to be resolved in the field. Advancements in these areas would not only push the CD8 T cell field forward but also provide insights into human disease pathogenesis on a more general level.

### Reconciling exhaustion, tissue residency, and effector function profiles

As discussed above, there is currently a degree of ambiguity and overlap between exhaustion, tissue residency, and effector function programs in CD8 T cells. Do these terms define distinct cell subsets, or is it more accurate to describe some or all of these terms as phenotypes? The difference between cell phenotype and cell subset is sometimes more philosophical than biological, but a better understanding of how current CD8 T cell subsets intersect would be helpful to the field. For example, CD8 T cells in tissues can express CD103 and CD69 alongside inhibitory receptors, and these cells have reduced inflammatory effects compared to other CD8 T_RM_ cells. Are these cells CD8 T_RM_ cells or T_EX_ cells or both? Have CD8 T_RM_ cells that express inhibitory markers and reduced effector functions received the same types of signals as T_EX_ cells that lack markers of tissue residency? Similarly, CD8 T_PEX_ cells often express GZMK, but not all GZMK+ CD8 T cells exhibit markers of exhaustion. What signals induce a CD8 T cell to express GZMK? Recent reports point to a role for IL-15 (148, 149), but the full spectrum of signaling pathways that govern GZMK expression remain undefined. Have all GZMK+ CD8 T cells at one point been a T_PEX_ cell, or do T_PEX_ cells differentiate from non-exhausted GZMK+ CD8 T cells? Are GZMK+ CD8 T cells and GZMB+ CD8 T cells always largely distinct populations? Current datasets focused on skin and synovium suggest they are (1, 201, 202), but it’s unclear if these findings hold true across all tissues and diseases. Single-cell transcriptomic studies that include TCR sequencing may be useful for investigating these questions, using TCR sequences as barcodes to link T cells that originate from the same parental cell. *In vitro* studies of human cells and *in vivo* mouse models may also be helpful.

### Bystander vs antigen-specific CD8 T cells

CD8 T cells can be activated independently of TCR-mediated stimulation by cognate peptide antigens, either through cytokine signaling (e.g., IL-15 or IL-18) or via engagement of stimulatory receptors such as NKG2D. This is called bystander activation, and it is likely a common occurrence at sites of inflammation (287, 288). Bystander activation is often helpful in fighting off infections. For example, in acute viral lung infection, bystander CD8 activation through type I interferon signaling results in high GZMB expression, enhanced antiviral cell killing, and decreased viral loads (289). Bystander CD8 T cells also assist with the control of *Burkholderia pseudomallei* and *Listeria monocytogenes* through production of IFNγ (290).

However, bystander CD8 T cells can be harmful if they amplify immune responses to the point that surrounding cells and tissues sustain damage beyond what is needed to block the infection or tumor. Bystander-activated GZMB+ CD8 T cells are implicated in several tissue immunopathologies including skin lesions in *Leishmania* infection (291) and hepatocyte damage in acute hepatitis A (292) and chronic hepatitis C (293). In a mouse model of ZIKA virus infection, bystander-activated NKGD2+ CD8 T cells correlate with neurological disease, and depletion of CD8 T cells is protective from ZIKV-associated paralysis (294).

Estimating the frequency and phenotypes of bystander CD8 T cells in human immune responses is very challenging since the identity of all relevant peptide antigens is rarely known, ruling out MHC class I tetramers as a viable approach to comprehensively identify antigen-specific and bystander CD8 T cells. Many common activation markers, such as CD69 and HLA-DR, increase with both cytokine and TCR-mediated stimulation and thus cannot differentiate TCR stimulation from bystander activation (72). Markers such as CD38 and CD39 have been suggested to indicate antigen-specific T cells in tissues, but other studies have found that cytokine signals regulate expression of these markers as well, leading to doubts about their sensitivity and specificity for identifying antigen-reactive cells (295–298). In short, bystander CD8 T cells are likely potent participants in immune responses, with potentially greater effects than antigen-specific CD8 T cells. Additional studies are needed to identify markers of these cells in human tissues and investigate their functional contributions, especially in cancers and autoimmune diseases.

### Manipulating CD8 T cells for therapeutic purposes

Immune checkpoint inhibitors are widely used and highly effective treatments for many cancers. However, many patients experience immune-related adverse events (irAEs) during ICI treatment, including inflammation of the skin or gastrointestinal tract, thyroid disease, or inflammatory arthritis (299–301). Are these side effects unavoidable, or is there a way to target ICIs more specifically to activate only antigen-specific anti-tumor T cells or, alternatively, all tumor-infiltrating T cells (whether antigen-specific or bystanders)? Answering this question will require deeper characterization and comparison of tumor-infiltrating T cells compared with T cells in other tissues, especially tissues prone to irAEs.

Similarly, chimeric antigen receptor (CAR) T cells are currently primarily made *ex vivo* and then re-infused into patients, but *in vivo* CAR-T constructs are approaching clinical use (302). *In vivo* treatments involve intravenous infusions of viral vectors or lipid nanoparticles that deliver transcripts encoding a relevant CAR to all T cells in the body. Depending on the CAR platform (retrovirus versus RNA delivered by lipid nanoparticle), the T cells express the CAR for either days to weeks (RNA) or months (retrovirus), after which the T cells persist but without the CAR (302). How does transient expression of a potent activating receptor change the baseline behavior or these cells? Are they more likely to become autoreactive over time? The primary goal of CAR-T cell studies is the design of new, effective anti-cancer treatments, but they will also provide useful insights into T cell biology and self-tolerance.

## Conclusion

CD8 T cells are common in blood and enriched in tissues in many disease settings, and they have the potential to perform a diverse array of effector functions. The growing number and size of transcriptomic datasets from cancers, infections, and autoimmune diseases provides us with a new and unbiased view of CD8 T cell populations and phenotypes in blood and tissue. To leverage these datasets into new insights and treatments, investigators must accurately identify CD8 T cell subsets and understand the spectrum of effector functions each CD8 T cell subset can perform.

With this goal in mind, we have provided a concise guide to CD8 T cell subsets in blood and tissue, including markers and effector functions. We discussed areas of overlap between populations, and we have also presented a simplified classification method using GZMB and GZMK expression to easily identify cytotoxic versus non-cytotoxic pro-inflammatory cells in large translational human -omics datasets. GZMB- and GZMK-associated CD8 T cell subsets are associated with many different diseases and with both positive and negative responses to treatment. The mechanisms of these associations remain unclear, but given the potency and prevalence of these CD8 T cell subsets, they inspire hope for new treatment approaches.

For decades, CD8 T cells were considered a monolithic cytotoxic population, but in recent years, our understanding of CD8 T cell phenotypes and subsets has broadened into a complex network of populations and effector functions. This expanded view of the range of CD8 T cell biology is an opportunity to re-evaluate the functions of these cells in each specific tissue and disease context. Accurate identification of CD8 T cell phenotypes and subsets in -omics datasets is the first step to understanding the function of CD8 T cells in blood and tissues in both health and disease.
